# 超声辅助气液微萃取-气相色谱-质谱法测定海带中16种多环芳烃

**DOI:** 10.3724/SP.J.1123.2025.01030

**Published:** 2025-08-08

**Authors:** Zhiguo MU, Yixuan WANG, Yanlin GUO, Xiangzi JIN, Donghao LI, Jinhua ZHAO

**Affiliations:** 1.延边大学理学院化学系，农产品质量与安全评价吉林省高校重点实验室，吉林 延吉 133002; 1. Department of Chemistry，College of Science，Yanbian University，Key Laboratory of Agrifood Quality and Safety Evaluation of Jilin Province，Yanji 133002，China; 2.延边大学分析测试中心，吉林 延吉 133002; 2. Analysis and Inspection Center，Yanbian University，Yanji 133002，China

**Keywords:** 超声辅助, 气液微萃取, 气相色谱-质谱法, 多环芳烃, 海带, ultrasound-assisted（UA）, gas-liquid microextraction（GLME）, gas chromatography-mass spectrometry（GC-MS）, polycyclic aromatic hydrocarbons（PAHs）, seaweed

## Abstract

基于超声辅助气液微萃取（UA-GLME）联用气相色谱-质谱（GC-MS）技术，建立了测定海带中16种多环芳烃（PAHs）的检测方法，并对样品前处理和色谱条件进行了优化。海带样品用二氯甲烷-正己烷（1∶1，v/v）混合溶剂超声辅助提取后，经过气液微萃取技术进一步萃取，采用DB-5MS毛细管柱（30 m×0.25 mm×0.25 µm）分离，使用全扫描（SCAN）、选择离子监测（SIM）模式进行检测分析。结果表明，本方法在最优的条件下，PAHs在5.0~2 000 ng/mL范围内具有良好的线性关系，相关系数（*R*^2^）均大于0.999，仪器检出限（LOD）和方法检出限（MDL）分别为0.001~0.01 μg/mL和0.004~0.04 mg/kg。16种PAHs在高、中、低3个水平下的加标回收率为62.32%~91.64%，RSD为2.94%~9.15%。本方法显著减少了脂溶性和共萃取物的干扰，其中长链烷烃、脂肪酸及甾醇类等主要共萃取物的去除率大幅提升。SIM模式下色谱图的比较分析结果表明，UA-GLME方法显著优化了PAHs的峰形，有效提高了信噪比和定量分析的准确性，同时展现出对海带基质中复杂干扰物的显著抗干扰能力。采用所建立的方法对东海、渤海、黄海及南海海带样品进行分析测定，结果显示16种PAHs均有不同程度的检出。该方法简化了复杂的样品前处理过程，并适用于海带中PAHs的定性、定量分析，为海洋环境污染和风险评价提供了可靠的技术支持。本方法可通过优化超声条件与溶剂体系，拓宽在其他复杂基质样品中的适用性，为环境监测与污染物溯源提供新策略。

## Abstract (en)

The increasing demand for the detection of polycyclic aromatic hydrocarbons（PAHs） in the complex matrices of the marine environment requires overcoming the limitations of traditional detection methods， particularly in terms of sensitivity and accuracy. Analyzing PAHs in seaweed matrices is often hindered by significant interference owing to the complex compositions of these matrices， which compromises both qualitative identification and quantitative determination. To address these challenges， this study developed an innovative and efficient method for detecting PAHs based on ultrasound-assisted gas-liquid microextraction（UA-GLME） coupled with gas chromatography-mass spectrometry（GC-MS）. The developed method involves extracting a seaweed sample with dichloromethane-hexane（DCM-HEX）（1∶1， v/v）， followed by further GLME. The separated sample is then analyzed using a DB-5MS capillary column（30 m×0.25 mm×0.25 µm） in SCAN and selective ion monitoring（SIM） modes. PAH extraction efficiencies were enhanced while significantly minimizing the co-extraction of interfering compounds by exploiting the cavitation effects of ultrasound. This optimization procedure improves both PAH-detection sensitivity and accuracy while reducing matrix effects. Under optimal conditions， the PAHs showed excellent linearity in the range of 5.0-2 000 ng/mL， with correlation coefficients（*R*^2^） exceeding 0.999. Limits of detection（LODs） ranged between 0.001 and 0.01 μg/mL， while method detection limits（MDLs） of 0.004–0.04 mg/kg were recorded. The recovery rates at high， medium， and low concentration levels were 62.32%-91.64%， with relative standard deviations（RSDs） between 2.94% and 9.15%. The developed method significantly reduces interference from lipophilic and low-volatility co-extractives， while removing co-extractives， such as long-chain alkanes， fatty acids， and sterols， at rates of up to 100%. Chromatographic comparisons revealed that the UA-GLME method delivers superior peak shapes for the target PAHs， enhanced signal-to-noise ratios， high quantitative accuracies， and remarkable resistance to seaweed-matrix interference. The method was used to detect PAHs in seaweed samples from the East China Sea， Bohai Sea， Yellow Sea， and South China Sea， with the 16 PAHs detected at varying levels. Both low-molecular-weight PAHs（such as naphthalene， phenanthrene， and fluorene） and high-molecular-weight PAHs（such as benzo［*a*］pyrene and benzo［*k*］fluoranthene） were detected at high rates. This method offers a reliable approach for the qualitative and quantitative analysis of PAHs in seaweed， thereby providing valuable technical support for assessing marine environmental pollution and evaluating risks. The developed method can be extended to other complex matrices by optimizing the ultrasound conditions and solvent system， thereby offering new strategies for environmental monitoring and pollutant-source tracing.

海带作为一种重要的海洋食品资源，因其富含矿物质、膳食纤维和生物活性物质，在全球膳食结构中占据重要地位。然而，近年来，随着工业排放、石油泄漏和沿海开发的增加，海洋环境中的多环芳烃（PAHs）污染问题日益严峻^［1］^。PAHs是一类具有持久性和生物累积性的有机污染物，其主要来源包括石油产品燃烧、工业废水排放以及大气沉降^［2］^。研究表明，海洋生态系统中的PAHs可通过水体沉积、食物链传递等途径进入海洋生物体，并在海产品如海藻类中发生富集，成为PAHs在海洋生态系统中的重要富集介质和指示生物^［3，4］^。据文献报道，在全球范围内，PAHs的高浓度现象在沿海区域尤其显著，如中国渤海、珠江口等地的水体和沉积物中检测出高浓度的PAHs，且其生物累积效应对海洋生物造成潜在的生态毒性^［5］^。此外，海产品的消费安全性受到广泛关注，研究表明，苯并［*a*］芘已在部分海带样品中被检出且含量超出食品安全限值，具有较高的致癌风险^［6，7］^。因此，以海带中PAHs作为检测对象监测海洋环境污染具有重要意义。

对海带等海洋植物中的多环芳烃进行检测时，基质的复杂性对分析结果的干扰是一个关键挑战。海带富含多种天然成分，如多糖、蛋白质、脂类及无机盐，这些成分在色谱分析过程中可能与目标化合物产生共萃取效应，导致基质效应的发生，从而影响气相色谱-质谱（GC-MS）的定性和定量分析^［8，9］^。研究表明，样品中复杂基质成分可能会造成离子抑制或信号增强，影响分析物的回收率和灵敏度^［10］^。基质的主要干扰来源包括脂溶性高且挥发性较低的共萃取物，这些物质往往无法在气相色谱进样过程中完全汽化，从而在进样口或色谱柱上残留，导致峰拖尾、基线漂移，甚至影响分析物的保留时间和响应强度^［11］^。例如，某些脂类和复杂碳水化合物会在分析过程中与PAHs产生共流出现象，干扰其准确定量。此外，共萃取的非挥发性极性化合物，如糖类和蛋白质，会影响离子源的稳定性，导致信号抑制。在海带样品中进行PAHs分析时，萃取溶剂的选择至关重要。仅依靠溶剂的极性来优化萃取过程可能无法充分去除基质干扰物，需综合考虑溶剂的极性和沸点，以避免脂溶性强、挥发性低的共萃取物残留在色谱系统中^［12，13］^。

目前，基质效应的常见消除方法包括凝胶渗透色谱（GPC）^［14］^、固相萃取（SPE）^［15］^和液相萃取（LPE）^［16，17］^等技术。GPC基于分子尺寸的分离原理，能够去除蛋白质、脂肪等大分子干扰物，但对脂肪酸、色素等小尺寸干扰物的去除效果有限。SPE依赖目标物、流动相与吸附剂的极性差异，通过溶解度变化实现分离，然而其净化能力有限，可能影响检测灵敏度和可靠性。LPE利用目标物在不同溶剂中的溶解度差异进行分离，尽管适用范围广，但通常需要大量溶剂，增加成本和操作复杂性。此外，微萃取作为一种集取样、萃取和浓缩于一体的前处理技术，依托固相或液相的物理化学性质选择性富集目标物，能够有效简化前处理过程，因而广泛应用于多种分析任务^［18］^。然而，这些传统方法均依赖于尺寸、相溶性和分子间相互作用来实现目标物的萃取与净化，对于脂溶性强、沸点高的污染物，其去除效率往往有限，难以彻底消除基质干扰。此外，高脂基质中常见的顽固性共提取物，可能在气相色谱进样口或色谱柱上积聚，导致分析结果的偏差和检测灵敏度的降低。

气液微萃取（GLME）是一种集样品萃取与净化于一体的高效前处理技术，特别适用于复杂基质样品中痕量污染物的测定^［19］^。GLME的基本原理依赖于目标分析物与基质干扰物在气液界面的传质行为，利用惰性气体的流动将样品中的挥发性和半挥发性化合物从液相中迁移至气相，再在低温条件下将其富集至微量有机溶剂中进行后续分析。相比于传统的萃取方法，GLME的主要特点在于其能够利用目标物与基质组分的沸点差异，实现高选择性的萃取。前期研究发现，该技术对脂溶性强、沸点高的共萃取物（如脂肪酸、蜡质等）具有良好的排除效果，有效减少色谱柱污染和峰拖尾问题，确保分析结果的准确性和重现性^［12］^。在复杂基质样品萃取分析时，混合有机溶剂的选择增强了萃取效率和净化能力^［20］^，而且超声辅助气液微萃取技术（UA-GLME）中超声波的空化效应可以加速高脂肪动物源食品中农药从基质中的释放，提升萃取效率，并降低共萃取物的引入^［21］^。

本研究基于UA-GLME联用GC-MS技术，建立了一种测定海带中16种多环芳烃的方法。该方法克服了传统萃取方法难以消除海带样品中脂肪酸、糖类及色素等难挥发性共萃取物的缺点，最大程度降低了基质效应对GC-MS分析的影响，提高了检测的灵敏度、选择性和准确性，可为环境污染和风险评估提供技术支持。

## 1 实验部分

### 1.1 仪器、试剂与材料

气相色谱-质谱联用仪（GC-MS-QP2010 Ultra，日本岛津公司）、冷冻干燥机（美国VirTis公司）、试管混合器（日本亚速旺公司）、粉碎机（天津泰斯仪器有限公司）、离心机（LX-400，海门市其林贝尔仪器制造有限公司）、AS系列超声波清洗机（天津奥特赛恩斯仪器有限公司）、超纯水系统（Milli-Q IQ7000，美国Millipore公司）。

16种PAHs标准品：萘（Nap）、苊（Ace）、苊烯（Acy）、芴（Flu）、菲（Phe）、蒽（Ant）、荧蒽（FluA）、芘（Pyr）、苯并［*a*］蒽（BaA）、䓛（Chr）、苯并［*b*］荧蒽（BbF）、苯并［*k*］荧蒽（BkF）、苯并［*a*］芘（BaP）、茚并［1，2，3-*cd*］芘（InP）、二苯并［*a，h*］蒽（DBahA）、苯并［*g，h，i*］苝（BghiP），以及2种同位素替代内标：荧蒽-d_10_（Flu-d_10_）、芘-d_12_（Pyr-d_12_），1种仪器内标菲-d_10_（Phe-d_10_），均购自美国Supelco公司。

本实验中所使用的海带样品均购于延吉，以不同产地为区分，主要包括东海、渤海、黄海和南海，每个产地购买3份。二氯甲烷（DCM）和正己烷（HEX）均为色谱纯，购自美国Thermo Fisher公司。

### 1.2 标准溶液的配制

准确称量标准品，用正己烷充分溶解得到PAHs标准储备液，于4 ℃冰箱保存。分别准确移取16种PAHs标准储备液于进样瓶中，用正己烷稀释，配制成质量浓度为5 mg/mL的混合标准工作液，涡旋混匀后于4 ℃冰箱保存。根据实验需要，用正己烷配制成不同浓度的系列混合标准工作液，现用现配。

准确称量同位素内标标准品，加入正己烷溶解，得到内标储备液，于4 ℃冰箱保存。分别精密移取3种同位素内标储备液于容量瓶中，用正己烷稀释，配制成10 μg/mL芘-d_12_和1 μg/mL荧蒽-d_10_的替代内标工作溶液和1 μg/mL菲-d_10_的仪器内标工作溶液。工作液于4 ℃冰箱封口保存，现用现配。

### 1.3 样品前处理

将海带样品剪成小块，真空冷冻干燥24 h，使用粉碎机将其研磨，用60目小筛过筛后经研钵研磨成粉状。

准确称取200 mg粉末样品，加入20 µL 10 μg/mL的芘-d_12_，再加入380 µL的二氯甲烷-正己烷（1∶1，v/v），涡旋混匀，在20 ℃下超声提取5 min，以2 500 r/min离心10 min，取上清液100 µL，加入40 µL的1 μg/mL的荧蒽-d_10_，经过气液微萃取仪萃取，接收相以及洗脱液均为20 µL的二氯甲烷-正己烷（1∶1，v/v），萃取后的液体样品加入10 mg无水Na_2_SO_4_除水，然后加入20 µL 1 μg/mL的菲-d_10_，待GC-MS分析。

### 1.4 分析条件

#### 1.4.1 色谱条件

色谱柱：DB-5MS毛细管柱（30 m×0.25 mm×0.25 µm）；色谱柱升温程序：初始温度80 ℃，保持2 min；以20 ℃/min的速率升高至100 ℃；以10 ℃/min的速率升高至200 ℃；以20 ℃/min的速率升至280 ℃，保持14 min。载气：氦气，纯度≥99.999%；流速：1.0 mL/min；进样口温度：280 ℃；进样体积：1 μL；进样方式：不分流进样。

#### 1.4.2 质谱条件

采用电子轰击源（EI），轰击能量：70 eV；离子源温度：200 ℃；溶剂切割时间：6 min；扫描方式：全扫描（SCAN）结合选择离子监测（SIM）模式。样品提取液中共萃取物的定性使用NIST 23数据库。其他质谱参数见表1。

**表 1:** 16种PAHs及3种同位素内标的质谱参数

Compound	Retention time/min	Quantitative ion（*m/z*）	Qualitative ions（*m/z*）
Naphthalene（Nap）	6.683	128	127/129
Acenaphthylene（Acy）	10.250	152	151/150
Acenaphthene（Ace）	10.667	154	153/152
Fluorene（Flu）	11.908	166	165/163
Phenanthrene（Phe）	14.093	178	176/179
Anthracene（Ant）	14.200	178	176/179
Fluoranthene（FluA）	16.192	202	200/101
Pyrene（Pyr）	16.533	202	200/101
Benz［*a*］anthracene（BaA）	18.483	228	226/229
Chrysene（Chr）	18.550	228	226/229
Benzo［*b*］fluoranthene（BbF）	21.089	252	250/253
Benzo［*k*］fluoranthene（BkF）	21.178	252	250/253
Benzo［*a*］pyrene（BaP）	22.134	252	250/253
Indeno［1，2，3-*cd*］pyrene（IcdP）	27.161	276	138/277
Dibenz［*a，h*］anthracene（DBahA）	27.402	278	279/139
Benzo［*ghi*］perylene（BghiP）	28.645	276	138/137
Phenanthrene-d_10_（Phe-d_10_）	14.092	188	189/184
Fluoranthene-d_10_（Flu-d_10_）	16.190	212	202/213
Perylene-d_12_（Pyr-d_12_）	22.339	264	260/265

## 2 结果与讨论

### 2.1 质谱条件优化

本实验采用DB-5MS毛细管色谱柱对目标物进行分离，分别通过SCAN和SIM模式进行定性和定量测定，选择丰度高、干扰低的特征离子为定量离子，丰度较高、干扰较低的2个特征离子作为定性离子。16种PAHs及3种内标混合标准溶液（1 μg/mL）的总离子流色谱图见图1。

**图 1:**
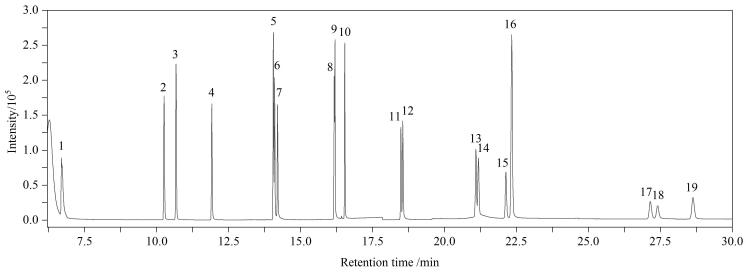
16种PAHs及3种内标混合标准溶液（1 μg/mL）的总离子流色谱图 1. Nap； 2. Acy； 3. Ace； 4. Flu； 5. Phe-d_10_； 6. Phe； 7. Ant； 8. Flu-d_10_； 9. FluA； 10. Pyr； 11. BaA； 12. Chr； 13. BbF； 14. BkF； 15. BaP； 16. Pyr-d_12_； 17. IcdP； 18. DBahA； 19. BghiP.

### 2.2 萃取溶剂的优化

萃取溶剂种类的选择会直接影响海带样品中PAHs的萃取效率，因此对萃取溶剂进行了优化。鉴于PAHs具有高度脂溶性，本研究对比了DCM、HEX、DCM-HEX（1∶1， v/v）三者的萃取效率（见图2）。在使用DCM作为萃取溶剂时，16种PAHs的回收率范围为44.76%~79.88%，其较强的极性和溶解能力使其在萃取相对分子质量较低的PAHs（如Nap，回收率为72.19%）时表现出色，但对于相对分子质量较高的PAHs（如BghiP，回收率为64.97%）的萃取能力则相对较弱。相比之下，HEX作为萃取溶剂时，16种PAHs的回收率范围为45.54%~67.02%，其对脂溶性成分具有较好的相容性，但由于其极性较低，难以有效溶解较低极性的PAHs（如Flu，回收率仅为47.00%）。然而，当采用DCM-HEX（1∶1， v/v）混合溶剂时，PAHs的回收率显著提升，范围为62.31%~90.91%。尤其是对于相对分子质量较高的PAHs（如BaP，回收率为84.59%），混合溶剂展现出更为优异的萃取性能。因此，本研究选用DCM-HEX（1∶1， v/v）混合溶剂作为萃取溶剂。

**图 2:**
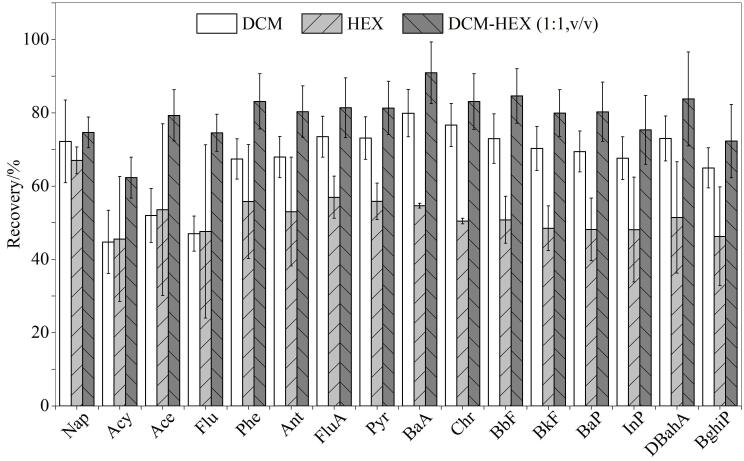
不同萃取溶剂种类对16种PAHs回收率的影响（*n*=3） HEX： hexane； DCM： dichloromethane.

### 2.3 萃取方式的考察

GLME技术通过有效提高复杂基质中干扰物与目标分析物的分离，保证目标物完全萃取的同时也可以对与目标物极性相似的难挥发性共萃取物进行高效净化^［22］^，以去除难挥发性物质对气相色谱分析时的干扰。对比色谱图可以发现，相比于GLME萃取的提取液（图3a），UA-GLME萃取后的提取液（图3b）中基质干扰峰显著减少，目标峰的分离度和对称性明显提高。采用GLME技术在海带萃取液中共检测到17种干扰物，包括长链烷烃（如二十烷（eicosane）、十五烷（pentadecane）、三十七烷（heptatriacontane））、脂肪酸及其衍生物（如十四酸（tetradecanoic acid）、十六酸（hexadecanoic acid）、亚油酸（linoleic acid）、花生四烯酸（arachidonic acid））、甾醇类（如醋酸豆甾醇（stigmasterol acetate）、Δ5-燕麦甾烯醇（delta 5-avenasterol））以及其他脂溶性成分（如2，3-环氧角鲨烯（2，3-epoxysqualene）、5*β*-孕甾-11-烯-3，20-二酮（5*β*-pregn-11-ene-3，20-dione））等。GLME萃取原理是在惰性气体保护的高温下，将待测物从样品基质中热解析出来，因此接收相中富集了海带基质中的挥发性和半挥发性物质。而UA萃取则通过调节溶剂的极性，有效减少了高疏水性干扰物的溶解。这一过程显著降低了共萃取物的种类和含量，同时消除了部分高疏水性化合物，从而提高了萃取的选择性和效率。

**图 3:**
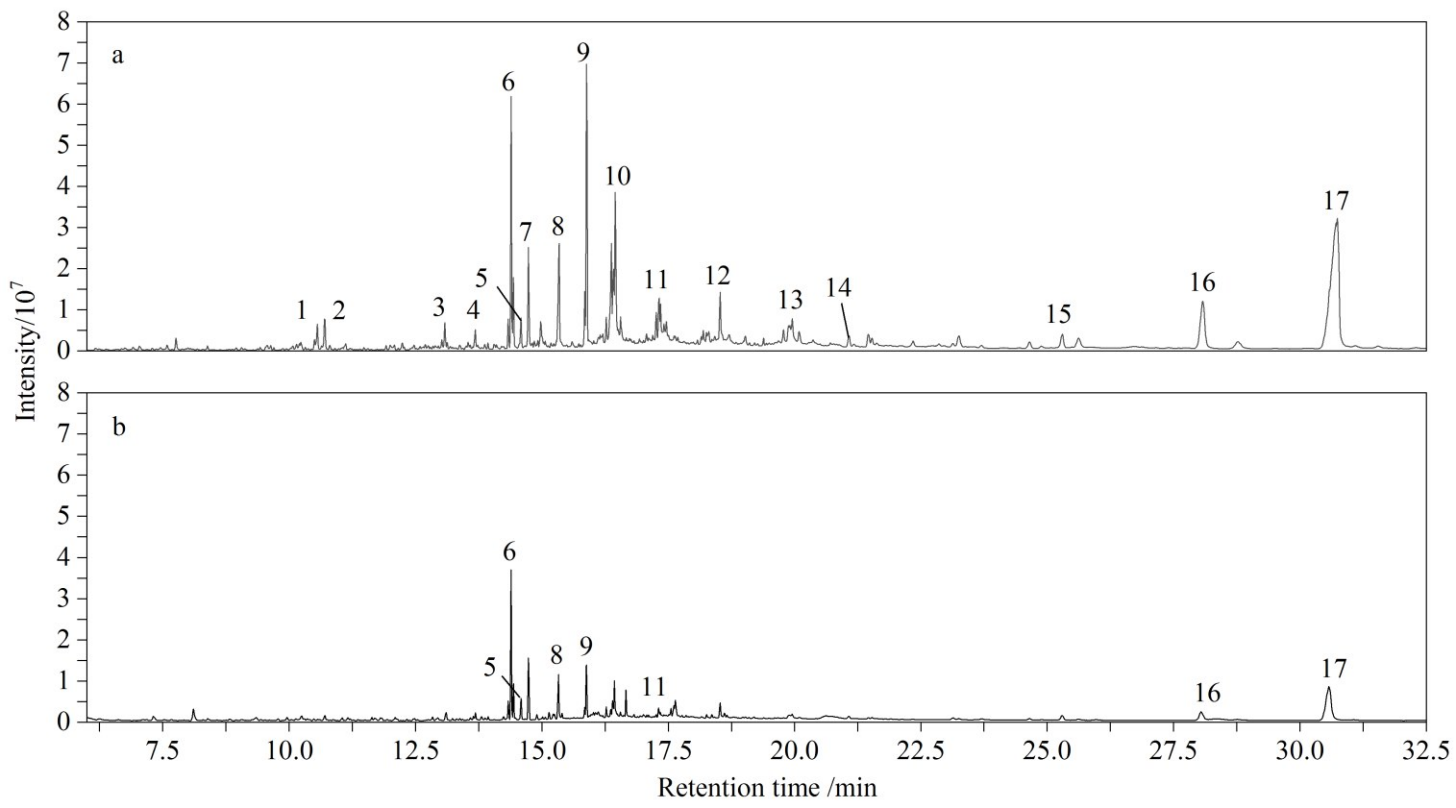
海带样品经（a）GLME与（b）UA-GLME萃取后的总离子流色谱图 GLME： gas-liquid microextraction； UA-GLME： ultrasound-assisted gas-liquid microextraction. Peak identifications： 1. eicosane； 2. pentadecane； 3. heptatriacontane； 4. tetradecanoic acid； 5. neophytadiene； 6. 3，7，11，15-tetramethyl-2-hexadecenyl acetate； 7. 3，7，11，15-tetramethyl-2-hexadecen-1-ol； 8. hexadecanoic acid； 9. docosapentaenoic acid methyl ester； 10. linoleic acid； 11. arachidonic acid； 12. 2-monopalmitin； 13.（5*Z*，11*Z*，14*Z*，17*Z*）-eicosatetraenoic acid methyl ester； 14. 2，3-epoxysqualene； 15. stigmasterol acetate； 16. 5*β*-pregn-11-ene-3，20-dione； 17. delta 5-avenasterol.

对海带样品中多环芳烃（加标量为20 ng）的色谱行为进行了超声辅助萃取效果的对比分析，以评估超声萃取对PAHs分离行为的影响。图4显示了超声萃取前后4种代表性PAHs的色谱图。结果显示，UA的引入有效减少了基质干扰物的共流出现象，并改善了目标化合物的峰形和信号强度，例如长链烷烃（二十烷，保留时间为10.52 min）的完全消除，有效改善了目标物在色谱柱上的分离性能，使得目标物的定量离子的响应（如Phe的*m/z* 178）在SIM模式下的更加稳定。以Phe、Ant和Pyr为例（图4），在未处理的GLME提取液中，峰形展宽且基线波动明显，主要受到相邻保留时间的高疏水性化合物的干扰，如二十烷、新植二烯（neophytadiene）等。这些干扰物因具有高log *P*值和低挥发性，在GLME过程中极易被共提取，从而增加了目标物定量分析的不确定性，还降低了脂溶性干扰物的萃取效率。特别是对于低挥发性化合物（如醋酸豆甾醇，保留时间为27.94 min）和长链脂肪酸（如十四酸，保留时间为14.36 min）在UA处理后的去除效果尤为显著，信号强度分别降低了82%和44%（图3）。这些改进显著减少了目标化合物邻近区域干扰物的共流出，从而显著提升了目标峰的对称性和分离度。例如，在芘和BghiP（图4）中，干扰物的去除显著提升了信号与背景噪声的比值（*S/N*），目标峰的轮廓变得更加清晰且重复性增强。这一结果进一步验证了UA与GLME联用在复杂基质样品分析中的准确性和定量分析的可靠性。

**图 4:**
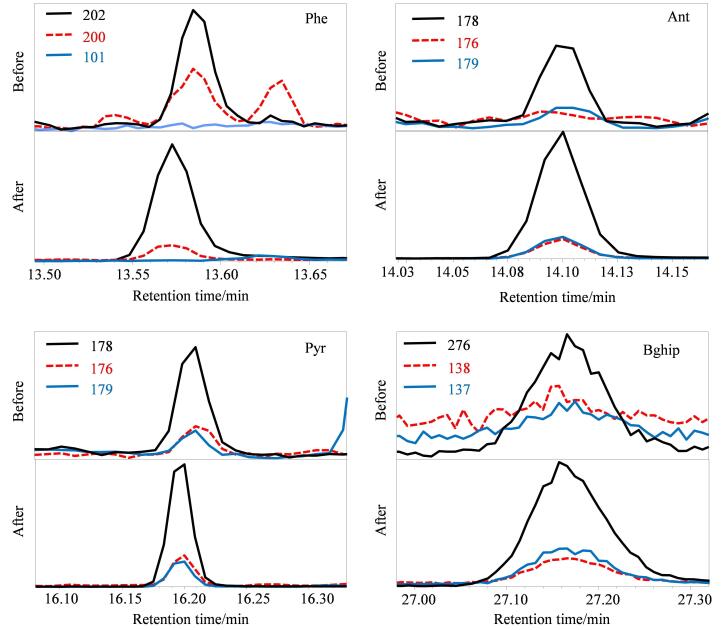
超声辅助萃取前后PAHs的色谱图

在定量分析方面，两种萃取方法均表现出良好的精密度和重复性，然而在PAHs的回收率方面，UA-GLME方法具有显著优势（见图5）。特别是相对分子质量的较低的PAHs（如萘、苊烯、芴等）和部分相对分子质量较高的PAHs（如苯并［*b*］荧蒽、苯并［*k*］荧蒽），回收率均在70%以上，最高可达90.91%。这一结果表明超声波的空化作用增强了溶剂与样品之间的接触和传质效率，增强液体流动，加速目标物质的高效释放，从而有效提高了PAHs的提取效率。

**图 5:**
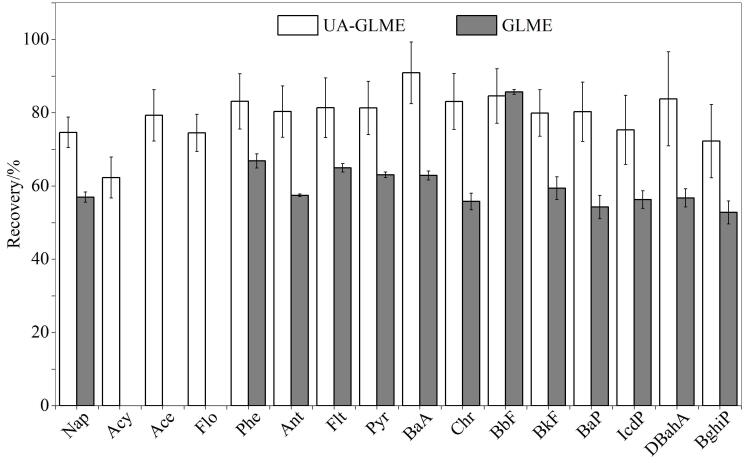
不同萃取方式对海带样品中PAHs回收率的影响（*n*=3）

综上所述，UA-GLME方法有效去除了脂溶性物质和共萃取物的干扰，显著提升了多环芳烃的检测灵敏度和定量准确性。该方法不仅优化了色谱分离效果，还在复杂基质样品中表现出优异的抗基质效应能力，为环境污染物分析和监测提供了技术支持。

### 2.4 方法学考察

#### 2.4.1 线性范围、检出限和方法检出限

对16种PAHs系列混合标准工作溶液（质量浓度为5.0~2 000 ng/mL）进行测定，以目标物与仪器内标峰面积的比值为纵坐标（*y*）、目标物与仪器内标质量浓度的比值为横坐标（*x*）绘制标准曲线。16种PAHs在5.0~2 000 ng/mL范围内表现出良好的线性关系，线性相关系数（*R* ^2^）均大于0.999。以*S/N*=3时的浓度作为仪器检出限（LOD），结合所取样品质量及最终定容体积，计算得出方法检出限（MDL）。本方法中16种多环芳烃的LOD为0.001~0.01 μg/mL，MDL为0.004~0.04 mg/kg（见表2）。

**表 2:** 16种PAHs的回归方程、相关系数、仪器检出限及方法检出限

Compound	Regression equation	*R* ^2^	LOD/（μg/mL）	MDL/（mg/kg）
Nap	*Y*=0.651*X*+2.412	0.9997	0.001	0.004
Acy	*Y*=0.562*X*+2.067	0.9996	0.001	0.004
Ace	*Y*=0.353*X*+1.475	0.9997	0.001	0.004
Flu	*Y*=0.353*X*+1.475	0.9998	0.001	0.004
Phe	*Y*=0.521*X*+1.241	0.9998	0.001	0.004
Ant	*Y*=0.521*X*+1.241	0.9999	0.001	0.004
FluA	*Y*=0.526*X*+1.292	0.9998	0.001	0.004
Pyr	*Y*=0.547*X*+1.271	0.9998	0.001	0.004
BaA	*Y*=0.510*X*-4.348	0.9995	0.002	0.008
Chr	*Y*=0.509*X*-6.931	0.9999	0.002	0.008
BbF	*Y*=0.521*X*-3.487	0.9998	0.005	0.02
BkF	*Y*=0.519*X*-2.380	0.9995	0.005	0.02
BaP	*Y*=0.500*X*-4.327	0.9995	0.005	0.02
InP	*Y*=0.439*X*-7.074	0.9990	0.010	0.04
DBahA	*Y*=0.365*X*-7.487	0.9985	0.010	0.04
BghiP	*Y*=0.471*X*-4.653	0.9996	0.010	0.04

Linear range： 5.0-2000 ng/mL； *Y*： peak area ratio of target compound to instrument intenal standard； *X*： mass concentration ratio of target compound to instrument internal standard.

#### 2.4.2 加标回收率和精密度

在最优条件下，向空白海带样品中添加低、中、高３个水平（0.05、0.50、2.0 μg/mL）的混合标准溶液考察回收率，每个水平分别测试3份平行样品。如表3显示，16种PAHs的加标回收率为62.32%~91.64%，RSD为2.94%~9.15%，均得到良好的回收率和精密度。

**表 3:** 海带中16种PAHs的加标回收率及相对标准偏差（*n*=3）

Compound	0.05 µg/mL		0.50 µg/mL		2.0 µg/mL	
	Recovery/%	RSD/%	Recovery/%	RSD/%	Recovery/%	RSD/%
Nap	72.68	4.13	74.65	2.96	85.54	5.06
Acy	63.21	5.83	62.32	3.95	67.94	6.51
Ace	86.91	8.39	79.29	4.96	91.64	4.66
Flu	74.35	5.73	74.52	3.59	82.80	4.28
Phe	84.91	7.19	83.11	5.35	85.29	8.33
Ant	78.68	5.47	80.33	4.96	77.71	2.94
FluA	87.27	6.19	81.38	5.77	82.17	6.81
Pyr	80.88	5.97	81.31	5.14	84.92	5.08
BaA	86.10	2.74	90.91	5.95	89.77	5.82
Chr	79.02	6.55	83.06	5.39	88.64	8.47
BbF	81.52	7.83	84.59	5.26	82.41	6.74
BkF	77.35	3.61	79.93	4.50	81.71	9.15
BaP	78.52	5.49	80.27	5.71	73.85	7.55
InP	69.83	8.45	75.33	6.66	76.69	6.77
DBahA	84.85	6.66	83.79	9.08	77.72	8.08
BghiP	85.63	5.67	72.28	7.08	75.44	7.90

### 2.5 方法对比

将本方法与其他文献报道的方法进行对比（见表4），本方法在分析样品量、有机溶剂消耗量以及分析时间等方面方法均有显著优势，本方法大幅度降低了有机溶剂和样品的使用量，实现了PAHs的高效定性定量分析，同时保持了良好的方法稳定性。

**表 4:** 本方法与文献报道方法的比较

Method	Samples	Sample amount	Extraction solvent volume/mL	Sample pretreatment time/min	Recovery/%	RSD/%
MEPS-GC-MS^［23］^	surface snow	4 mL	>4	>10	77.6-120.8	<15.00
LLE-SPE-GC-MS^［24］^	edible oils	500 mg	>15	>15	87-104	<7.50
Florisil-SPE-GC-MS^［25］^	biopolymers	100 mg	>30	>180	89-101	<18.00
QuEChERS-GC-MS^［26］^	poultry meat	5 g	>20	>13	71.2-104	<7.00
This work	seaweed	200 mg	0.4	10	62.32-91.64	≤9.15

MEPS： microextraction by packed sorben.

### 2.6 实际样品分析

将所建立的方法应用于不同海域海带样品中PAHs的测定，发现其检出情况存在显著差异，反映了海洋环境中PAHs污染的地域性特征。如表5所示，在东海、渤海、黄海及南海海带样品中16种PAHs均有不同程度的检出，其中萘、菲、芘和苯并［*a*］芘的检出率较高，而萘是最普遍检出的化合物，其含量范围为0.029 4~0.080 0 mg/kg，这表明PAHs在海带样品中的分布较为广泛，可能与其在海洋环境中的高持久性和水溶性相关。此外，苯并［*a*］芘和苯并［*k*］荧蒽等相对分子质量较高的PAHs在部分样品中亦有检出，尤其是在渤海和东海样品中，其含量分别为0.023 6~0.048 4 mg/kg和0.025 7~0.100 8 mg/kg，显著高于其他区域，这可能与沿海工业活动和船舶排放密切相关。相比之下，南海样品中大部分PAHs的检出水平较低，部分化合物低于方法检出限。值得注意的是，相对分子质量较低的PAHs（如萘、菲和芴）的检出率普遍较高，可能其较高的水溶性和挥发性使其在海带吸附过程中更易积累。

**表5:** 实际海带样品中16种PAHs的含量（*n*=3）

Compound	East China Sea			Bohai Sea			Yellow Sea			Southern China Sea
	No. 1	No. 2	No. 3	No. 1	No. 2	No. 3	No. 1	No. 2	No. 3	
Nap	0.0294	0.0788	0.0800	<MDL	0.0386	0.0292	0.0263	ND	0.0391	0.0338
Acy	ND	ND	0.0130	ND	ND	ND	<MDL	0.0225	<MDL	ND
Ace	ND	ND	ND	ND	ND	ND	ND	ND	ND	ND
Flu	<MDL	<MDL	0.0294	<MDL	<MDL	<MDL	0.0250	<MDL	ND	<MDL
Phe	<MDL	<MDL	0.0422	<MDL	<MDL	<MDL	0.0317	<MDL	<MDL	ND
Ant	<MDL	<MDL	0.0302	<MDL	<MDL	<MDL	0.0230	ND	<MDL	ND
FluA	ND	ND	ND	ND	ND	ND	0.0336	ND	ND	ND
Pyr	ND	ND	ND	ND	ND	ND	0.0344	ND	ND	ND
BaA	<MDL	0.0350	0.0727	<MDL	0.0350	ND	0.0474	ND	ND	ND
Chr	<MDL	0.0349	0.0678	<MDL	0.0280	ND	0.0415	ND	0.0523	ND
BbF	0.0241	0.0516	0.0965	<MDL	0.0434	0.0227	0.0466	<MDL	<MDL	<MDL
BkF	0.0219	0.0516	0.0922	<MDL	0.0422	0.0210	0.0459	<MDL	<MDL	<MDL
BaP	0.0257	0.0603	0.1008	<MDL	0.0484	0.0236	0.0491	<MDL	<MDL	<MDL
IcdP	0.0452	0.0893	0.1416	0.0275	0.0768	0.0421	0.0610	<MDL	<MDL	<MDL
DBahA	0.0864	0.1301	0.1867	0.0665	0.1199	0.0878	0.1183	0.0502	0.0639	0.0689
BghiP	<MDL	0.0909	0.1450	<MDL	0.0790	<MDL	0.0559	<MDL	ND	<MDL

ND： not detected.

## 3 结论

本研究建立了海带样品中多环芳烃的UA-GLME-GC-MS测定方法，降低了基质对目标化合物检测的干扰。本方法通过减少脂溶性共萃取物的溶解及难挥发性物质的干扰，显著降低了基质效应对色谱分离和定量分析的干扰，从而提升了检测灵敏度和准确性，为海洋环境监测提供了一种简便、绿色且可靠的样品前处理方法。
